# Tests and Numerical Study of Single-Lap Thermoplastic Composite Joints Bolted by Countersunk

**DOI:** 10.3390/ma15072386

**Published:** 2022-03-24

**Authors:** Jian Zhang, Xiuhua Chen, Aiqin Tian, Yin Fan

**Affiliations:** 1 School of Mechanical Engineering, Shanghai Jiao Tong University, Shanghai 200240, China; zhangjian970603@sjtu.edu.cn; 2 School of Aeronautics and Astronautics, Shanghai Jiao Tong University, Shanghai 200240, China; 3 School of Materials Science and Engineering, Shanghai Jiao Tong University, Shanghai 200240, China; tianaiqin@cqsf.com; 4 CRRC Qingdao Sifang Co., Ltd., Qingdao 266111, China

**Keywords:** thermoplastic composite, single-lap countersunk bolted joint, 3D elastoplastic damage model, bolt-tightening torque, clearance

## Abstract

Tensile tests were carried out to investigate the effect of stacking sequences on the bearing strength of single-lap thermoplastic composite countersunk bolted joints. A 3D elastoplastic model was built based on a plastic theory for numerical analysis. The damage initiation was judged based on LaRC05 criteria, and the damage propagation was described by using a nonlinear, gradual unloading method based on crack band theory. The accuracy of the present model was validated by comparing the numerical results to those from the tests. The test results showed that the effects of stacking sequences on the ultimate bearing strength and the 2% offset bearing strength are limited. Moreover, the numerical results depicted that the ultimate bearing strength and the 2% offset bearing strength reduce when the bolt-tightening torque or the bolt–hole clearance is increased.

## 1. Introduction

Owing to their superior features, such as high specific strength and stiffness, high specific moduli, damage tolerance, and environmental resilience, carbon-fiber-reinforced thermoplastic (CFRTP) composites have been widely used in the aerospace industry [1,2,3,4,5]. Composite components are divided into numerous parts as per the requirements of structural design, manufacture, and maintenance of composites, as well as the limitations of the integrity molding technology. As a result, the joining technique plays a key role in the application of composites [6]. Usually, fasteners are used to connect joint parts and bolted joints are frequently used in aircraft structures because they are easy to produce, disassemble, repair, and replace [7]. The bolted joints, however, are usually the weakest part in a composite laminate structure since a composite material is anisotropic and relatively brittle [8]. Therefore, the failure mechanism of composite joints is of most concern in the design of composite structures.

In the past decades, many scholars have conducted in-depth research into thermoset composite bolted joints [9,10,11,12,13,14,15,16,17,18,19,20,21,22,23,24,25]. For example, Park [11] investigated the influence of stacking sequences on the delamination bearing strength of mechanically fastened joints. Results revealed that the delamination bearing strength of the lay-up with the 90° layers on the surface was stronger than the one with the 90° layers located at the center of the laminate. Using experiments and 3D finite element models, McCarthy et al. [12,13] studied the influence of bolt–hole clearance on composite protruding bolted joints. They found that the increase of the clearance might lead the protruding bolts to rotate and the contact area and stiffness were both degraded as a result. Most scholars [11,12,13,14,15,16,17] have used 3D models to simulate composite bolted joints. To improve computational efficiency, Liu et al. [25] modified the bolt stiffness model based on the concept of control volume proposed by Gray and McCarthy [24]; Belardi et al. [23] proposed a novel composite bolted joint element. Both numerical methods had been adopted for accurately predicting the mechanical behaviors of composite bolted joints.

With the development of technology, thermoplastic composite materials have been increasingly used in modern industry and thermoplastic joints, such as pinned joints [7], riveted joints [26,27,28], and protruding bolted joints [29,30,31,32], have already been focused on. Yýlmaz and Sýnmazçelik [7] carried out experimental methods to investigate the bearing performance of the pinned joints of carbon fiber/polyphenylene sulfide (CF/PPS). The findings revealed that the bearing strength of the pinned joints improved if enough end distance and width were provided. Gay et al. [26,27] investigated the performance of a PA6.6-GF (glass fiber/polyamide)/aluminum assembly joined by self-piercing rivets under static and fatigue loads. They found that the domed head rivet achieved higher fatigue limit strength at 2 × 10^6^ cycles and higher bearing strength at static loading than that of the countersunk rivet and the failure mode under the fatigue load was determined by the load amplitude and the joint geometry. The influence of interference fit on the bearing strength of a carbon fiber/polyetheretherketone (CF/PEEK) riveted joint was investigated by Yao et al. [28] using both experimental and numerical approaches. According to their research, the extrusion between the rivet and the composite laminate created a plastic deformation on the hole wall, which would enhance the bearing capacity of the composite laminate; the acceptable interference-fit size was in the range of 2.0–2.6% for the CF/PEEK riveted joint. Vieille et al. [29,30,31] investigated the effect of environmental conditions on the mechanical behaviors of carbon-fiber-fabric-reinforced PPS laminates. It was found that the failure mode could be changed from brittle to progressive in a severe environment (120 °C after hygrothermal aging). The enhanced localized plasticization of the PPS and the degraded fiber/matrix interface dissipated a portion of the mechanical energy transmitted to the joint and contributed to easing the stress concentration, which led to an increases in the strength of the double-lap joints.

Although reports on mechanical behaviors of thermoplastic joints can be found, there is a shortage of studies on the tensile performance of thermoplastic composites with single-lap countersunk bolted joints. The thermoplastic composites made for single-lap countersunk bolted joints are first studied by tests in this paper, and then a 3D elastoplastic damage model that can properly replicate its mechanical behavior is proposed for analysis of their mechanical behaviors. The purpose of this paper is to reveal the failure mechanism and investigate the influence of bolt torque and bolt–hole clearance on the bearing strength of single-lap countersunk bolted joints of AS4/PEEK (carbon fiber/polyetheretherketone). Firstly, tensile tests of the single-lap countersunk bolted joints of laminates with three stacking sequences are performed to obtain load–displacement curves and failure modes. After that, a 3D elastoplastic model is developed based on a plastic theory proposed by Sun and Chen [33]. The LaRC05 criteria [34] are used to predict damage initiation, and a nonlinear gradual unloading method based on crack band theory [35] is used to simulate the damage extension. Then, the above 3D elastoplastic model, failure criteria, and damage propagation are all converted into a numerical simulation of single-lap countersunk bolted joints and this method is validated by comparing with the test results. Finally, the impacts of bolt-tightening torque and bolt–hole clearance on the ultimate bearing strength and the 2% offset bearing strength are investigated, respectively. The influence on the bearing strength of the single-lap countersunk joints is discussed in detail.

## 2. Test

### 2.1. Test Setup

**Figure 1:**
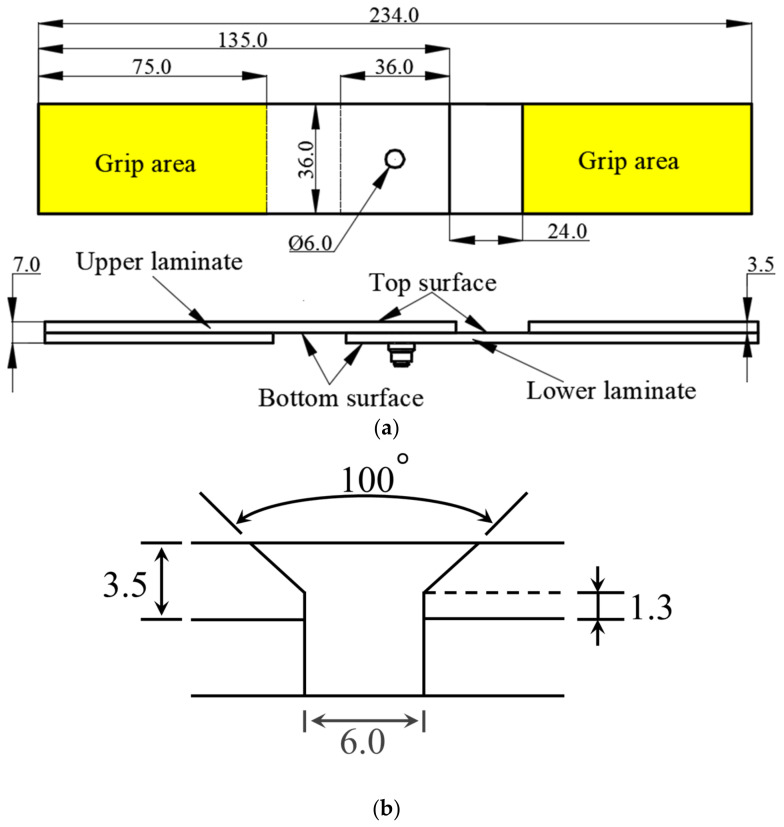
Geometry and dimensions (unit: mm) of the test specimen and the countersunk bolt: (**a**) test specimen and (**b**) countersunk bolt.

The dimensions of a test specimen are shown in Figure 1a, which are recommended by ASTM D5961/D 5961M-17 [36]. The specimens operated in the tests were manufactured by unidirectional AS4/PEEK prepregs, where the average fiber volume fraction was 59%. Each specimen had 25 layers, and the nominal thickness of each layer was 0.14 mm. There were three different stacking sequences, ${\left[-45/90/45/0/-45/0/45/0/-45/0/45/-45/\bar{90}\right]}_{s}$, ${\left[-45/90/45/0_{2}/-45/0/45/0/-45/0/45/\bar{90}\right]}_{s}$, and ${\left[-45/90/45/0_{2}/-45/0/45/0_{2}/-45/0/\bar{90}\right]}_{s}$, and the overbar represents the center layer in symmetric lay-up with an odd number of piles. The names of these three stacking sequences were DJ-A, DJ-B, and DJ-C. The width-to-diameter ratio was 6, and the end-distance-to-diameter ratio was 3. The 100° countersunk bolt used in the test specimen is made of Ti-6Al-4V and fixed with steel nuts. As shown in Figure 1b, the cylindrical depth of the countersunk bolt in the upper plate was 1.3 mm; the bolt-tightening torque was 1.8 N·m, and there was no clearance between the countersunk bolt and the laminate. To ensure neutrality during the test, pads with the same material as the test specimen were bonded at both ends of the test specimen, and the size of the pads was 75 mm × 36 mm × 3.5 mm. For each layup configuration, we tested six specimens.

**Figure 2:**
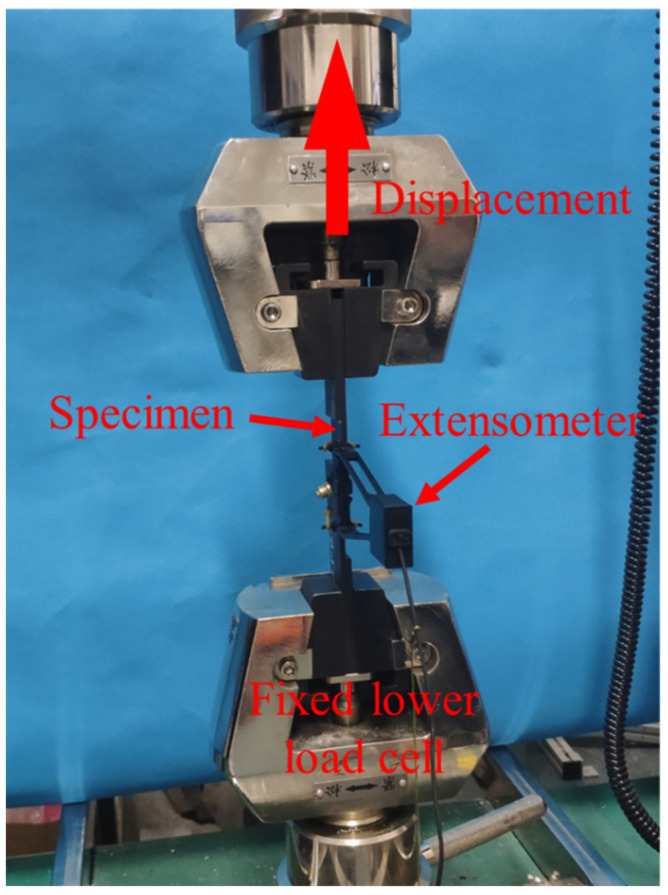
Test equipment of the AS4/PEEK countersunk single-lap bolted joint.

The test was carried out on the UTM5105X microcomputer—controlled electronic universal mechanical testing machine with 100 kN load capability. The laminates were clamped by the grip holder of the testing machine. The gripping area length was about 55 mm. An extensometer, was mounted on the specimen to measure the longitudinal hole deformation. The type of extensometer was SUNS-YSJ50/10 and the gauge distance was 50 mm. All above equipment was manufactured by Shenzhen SUNS Technology Stock CO., LTD. in Shenzhen, China. The test equipment is shown in Figure 2. The applied load was of a constant displacement rate of 2 mm/min, according to ASTM D5961/D 5961M-17 [36], up to the catastrophic failure. The data related to the applied load, grip holder displacement, and hole deformation were recorded automatically by the computer. After the tests, disassembling processes were executed to examine the damage in the laminates.

### 2.2. Test Results

**Figure 3:**
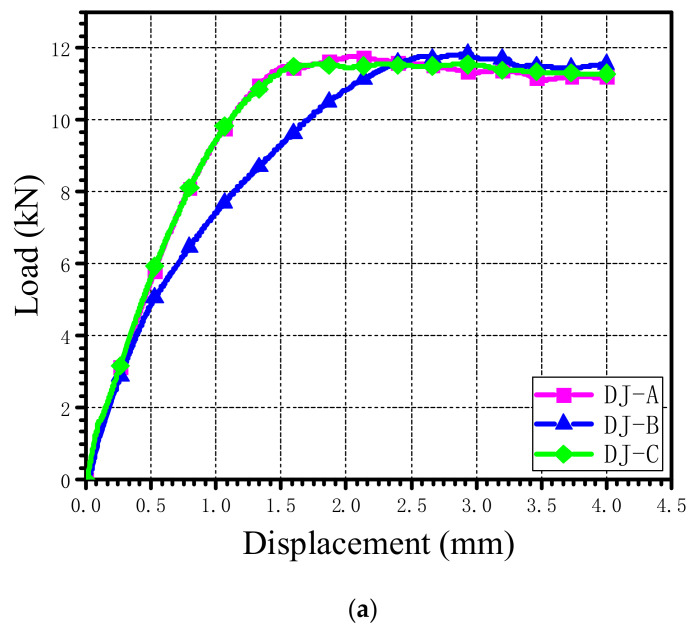

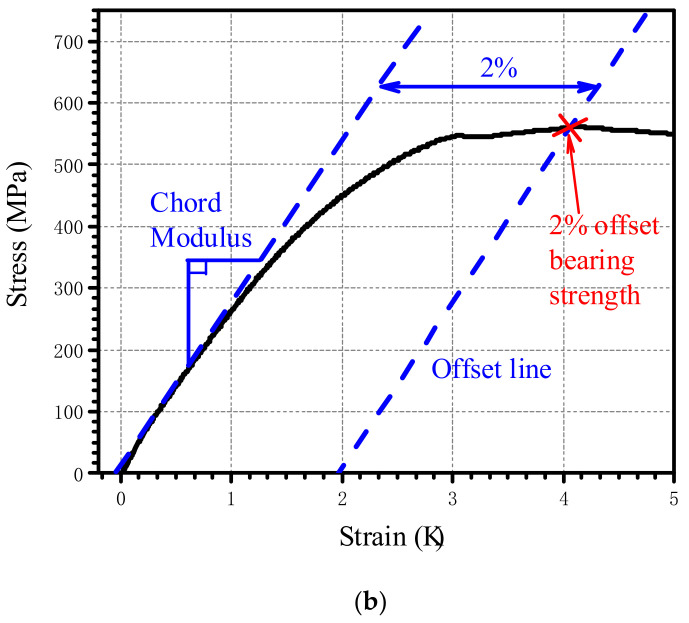
(**a**) Load–displacement curves of AS4/PEEK single-lap countersunk bolted joints with three stacking sequences. (**b**) Determination of the 2% offset bearing strength.

Figure 3a depicts one of the load–displacement curves obtained from the tests to demonstrate the mechanical responses of the countersunk single-lap bolted joints. The bearing strength and strain were calculated from the load–displacement data by using the equations $\sigma_{br}=F/\left(k\times D\times h\right)$ and $\varepsilon_{br}=\delta /\left(K\times D\right)$, where $\sigma_{br}$ and $\varepsilon_{br}$ are the bearing stress and strain, respectively; F and δ represent the load and the displacement measured during the bearing tests, respectively; D and h are the bolt hole diameter and the specimen thickness, respectively; k is the force per hole factor (1.0 for single-fastener or pin tests and 2.0 for double-fastener tests); and K is 2 in this test [36]. The 2% offset bearing strength was determined by the intersection point of the stress–strain curve and the translated chord stiffness line, as depicted in Figure 3b. The ultimate bearing strength and the 2% offset bearing strength corresponding to the test specimens are summarized in Table 1. The differences between the ultimate bearing strength of DJ-B and DJ-C and that of DJ-A were −0.33% and 0.70%, respectively, and the differences between the 2% offset bearing strength were 1.45% and −3.54%, respectively. It is apparent that the stacking sequences had a limited effect on the ultimate bearing strength and the 2% offset bearing strength of the single-lap countersunk joints.

**Figure 4:**
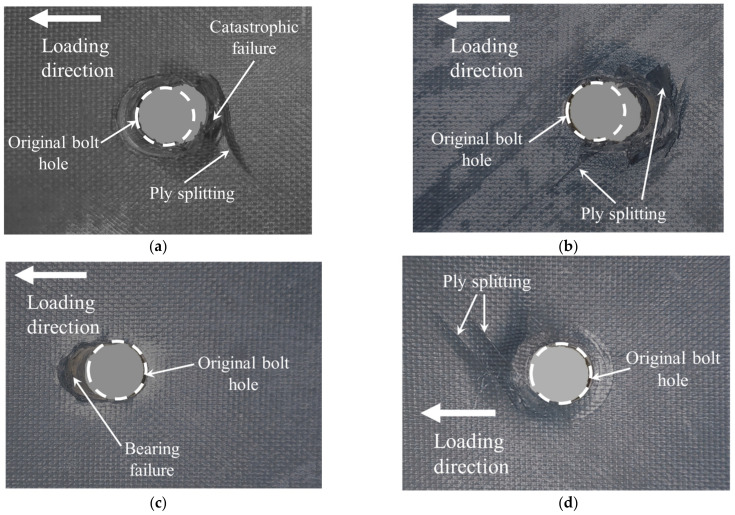

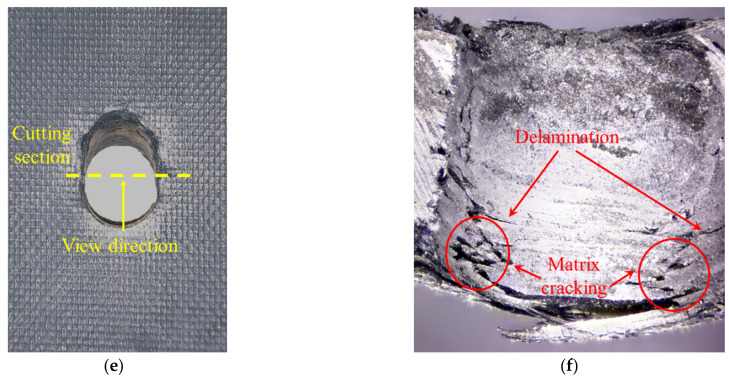
Damage to the specimens after tensile loading: (**a**) Upper laminate, top surface; (**b**) upper laminate, bottom surface; (**c**) lower laminate, top surface; (**d**) lower laminate, bottom surface; (**e**) cutting section and view direction; and (**f**) microscopic photograph.

The damage mainly occurred in the laminates, while no obvious damage occurred on the countersunk bolt. The photographs from different perspectives and the micrograph of the test specimen are shown in Figure 4. Around the bolt hole, we could see fiber-matrix splitting on both sides of the upper laminate and the bottom surface of the lower laminate. This damage is caused by the tensile bending stress arising from transverse loading [37]. The micrograph shows an evident compressive area in the top half of the lower laminate, whereas the lower section of the laminate was mainly the matrix cracking.

## 3. Numerical Model

### 3.1. Constitutive Relationship

**Figure 5:**
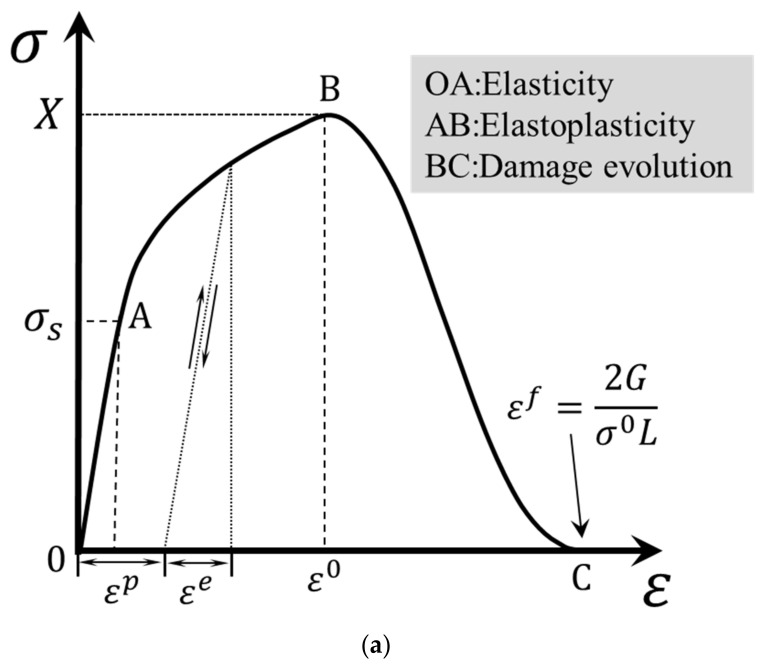

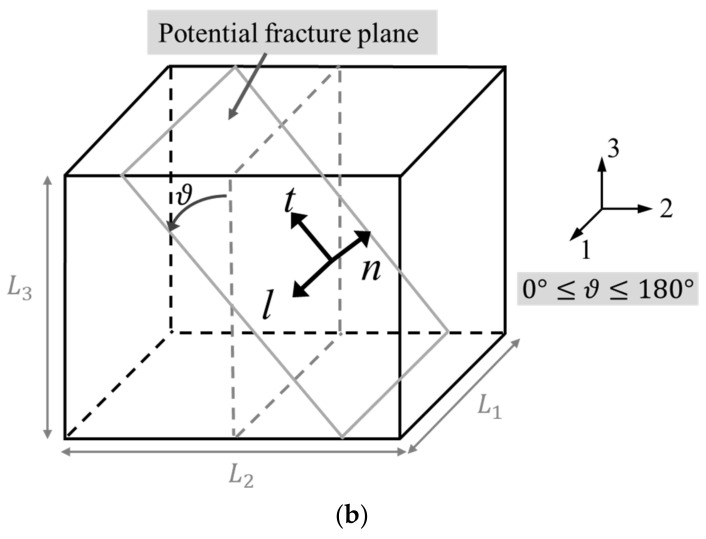
An illustration of (**a**) a typical stress–strain curve of elastoplastic materials, where X denotes the strength, and (**b**) calculation of tractions on any fracture plane.

For the unidirectional composite materials that exhibit plasticity and progressive degradation of material properties, this paper built a 3D elastoplastic damage model. It was developed for an elementary orthotropic ply and consisted of a plastic part that describes the plastic behavior of composites, failure criteria for predicting damage initiation, and damage evolution laws that account for the development of the failure process. The typical stress–strain curve of elastoplastic materials is shown in Figure 5a, which has three regions: an elastic region, an elastoplastic region, and a damage evolution region. Note that 1-direction is the longitudinal direction (fiber direction) and the 2-direction and 3-direction are the transverse directions in this paper.

#### 3.1.1. Elastic Model

Assuming that the laminate is homogeneous and orthotropic, the relationship between elastic stress and strain is formulated as:

(1) $$\sigma =C:\varepsilon^{e}=C:(\varepsilon -\varepsilon^{p}),$$

where σ is the stress tensor; C is the stiffness tensor; ε, $\varepsilon^{e}$, and $\varepsilon^{p}$ are the total strain tensor, the elastic strain tensor, and the plastic strain tensor, respectively; and the symbol (:) denotes the inner product of two tensors with double contraction. 

#### 3.1.2. Plastic Model

In this model, it is assumed that the plastic deformation occurs in the undamaged area of the laminates and the plastic flow rule and the hardening law are expressed in terms of effective stresses, equivalent plastic strain, and equivalent stress, which are based on the effective stress space concept [38].

The yield function used in this model is expressed as follows:

(2) $$F\left(\sigma ,\bar{\varepsilon^{p}}\right)=F^{p}\left(\sigma \right)-\kappa \left(\bar{\varepsilon^{p}}\right),$$

where $\bar{\varepsilon^{p}}$ is the equivalent plastic strain; $F^{p}\left(\sigma \right)$ is the plastic potential function; and κ is the hardening parameter, which depends on the plastic deformations and is expressed in terms of equivalent plastic strain. 

Due to its simplicity and accuracy, the 3D plastic potential function proposed by Sun and Chen [33] was adopted in this study. Weeks and Sun [39] assumed that the composite material is transversely isotropic in the 2–3 plane and linearly elastic in the fiber direction. Therefore, the plastic potential function is reduced to:

(3) $$\;F^{p}\left(\sigma \right)=\sqrt{\frac{3}{2}\left(\sigma_{22}^{2}+\sigma_{33}^{2}\right)-3\sigma_{22}\sigma_{33}+6\sigma_{23}^{2}+3a_{66}\sigma_{12}^{2}+3a_{66}\sigma_{13}^{2}},$$

where $a_{66}$ is a coefficient describing the amount of anisotropy in the plasticity. Its value can be determined using an approach based on the linear regression analyses of the off-axis tensile tests performed on the unidirectional composite laminates [33,40,41]. It should be noted that Figure 5a is only appropriate for describing the mechanical behaviors of non-fiber directions. The plastic strain increment can be calculated by the associated plastic flow rule:

(4) $$d\varepsilon^{p}=d\lambda \frac{\partial F^{p}}{\partial \sigma },$$

where dλ is a non-negative plastic consistency parameter. Let the equivalent stress be defined as:

(5) $$\bar{\sigma }=F^{p}.$$

The effective plastic strain increment $d\bar{\varepsilon^{p}}$ can be defined through the consideration of plastic work increment:

(6) $$dW^{p}=\sigma \cdot d\varepsilon^{p}=\bar{\sigma }d\bar{\varepsilon^{p}}.$$

Substitution of Equations (3)–(5) into (6) yields:

(7) $$d\bar{\varepsilon^{p}}=d\lambda .$$

Experimental results have proved that thermoplastic composites do not have a well-defined yield point [39]. For this reason, the yield stress $\sigma_{s}$ is always assumed to be 0 in many works [28,42,43,44,45]. An isotropic hardening function expressed by the equivalent plastic strain was adopted in this study:

(8) $$\kappa \left(\bar{\varepsilon^{p}}\right)=\beta {\left(\bar{\varepsilon^{p}}\right)}^{n},$$

where β and n are determined by fitting the experimental hardening curve. These values are shown in Table 2.

#### 3.1.3. Damage Initiation and Evolution Model

The maximum stress criteria were applied to predict the failure in the fiber direction:

(9) $$f_{ft}=\frac{\sigma_{11}}{X_{t}}\left(\sigma_{11}>0\right),$$

(10) $$f_{fc}=-\frac{\sigma_{11}}{X_{c}}\left(\sigma_{11}<0\right),$$

where $X_{t}$ and $X_{c}$ are the tensile and compressive failure strength in the fiber direction, respectively, and $f_{ft}$ and $f_{fc}$ are the fiber tensile damage index and the fiber compressive damage index, respectively. Fiber tensile failure will occur if $f_{ft}\geq 1$, and fiber compressive failure will occur when $f_{fc}\geq 1$.

To predict the failure initiation of a matrix-dominated failure, the LaRC05 criteria [34] were adopted in this study. The failure criteria assume that failure may appear on an arbitrary fracture plane parallel to the fiber direction depending on the combination of shear tractions ($\sigma_{nT}$ and $\sigma_{nL}$) and normal traction ($\sigma_{n}$) on the corresponding plane [46]. When the failure index on a certain fracture plane reaches 1, the composites fail on this plane. As shown in Figure 5b, the tractions on the potential fracture plane can be transformed from the material coordinate system using the following equations:

(11) $$\;\left\{\begin{array}{c} \sigma_{n}\left(\vartheta \right)=\frac{\sigma_{22}+\sigma_{33}}{2}+\frac{\sigma_{22}-\sigma_{33}}{2}\cos\left(2\vartheta \right)+\sigma_{23}\sin\left(2\vartheta \right)\\ \sigma_{nT}\left(\vartheta \right)=\frac{\sigma_{22}-\sigma_{33}}{2}\sin\left(2\vartheta \right)+\sigma_{23}\cos\left(2\vartheta \right)\\ \sigma_{nL}\left(\vartheta \right)=\sigma_{12}\cos\left(\vartheta \right)+\sigma_{13}\sin\left(\vartheta \right) \end{array}\right.,$$

where ϑ is the angle of the fracture plane, ranging from $0^{{}^{\circ}}$ to $180^{{}^{\circ}}$. In these criteria, two typical failure modes are considered, depending on the value of the normal traction, and the failure indexes are defined as:

(12) $$f_{mt}={\left(\frac{\sigma_{nT}\left(\vartheta \right)}{S_{T}-\mu_{T}\sigma_{n}\left(\vartheta \right)}\right)}^{2}+{\left(\frac{\sigma_{nL}\left(\vartheta \right)}{S_{L}-\mu_{L}\sigma_{n}\left(\vartheta \right)}\right)}^{2}+{\left(\frac{\sigma_{n}\left(\vartheta \right)}{Y_{T}}\right)}^{2}\left(\sigma_{n}\left(\vartheta \right)>0\right),$$

(13) $$f_{mc}={\left(\frac{\sigma_{nT}\left(\vartheta \right)}{S_{T}-\mu_{T}\sigma_{n}\left(\vartheta \right)}\right)}^{2}+{\left(\frac{\sigma_{nL}\left(\vartheta \right)}{S_{L}-\mu_{L}\sigma_{n}\left(\vartheta \right)}\right)}^{2}\left(\sigma_{n}\left(\vartheta \right)<0\right)\;,$$

(14) $$\;\mu_{T}=-\frac{1}{\tan\left(2\varphi_{0}\right)},S_{T}=\frac{Y_{c}}{2\tan\left(\varphi_{0}\right)},\mu_{L}=S_{L}\frac{\mu_{T}}{S_{T}},$$

where $S_{T}$ and $S_{L}$ are the transverse shear failure strength and the longitudinal shear failure strength, respectively; $\mu_{T}$ and $\mu_{L}$ are the transverse and longitudinal friction coefficients, respectively; $Y_{T}$ and $Y_{c}$ are the transverse tensile strength and the compressive failure strength of composite laminates, respectively; and $\varphi_{0}$ is the through-thickness direction under transverse compressive load. For carbon-fiber-reinforced composites, the value of $\varphi_{0}$ determined by experiments is $\varphi_{0}=53{}^{\circ}\pm 2{}^{\circ}$ [47]. $f_{mt}$ and $f_{mc}$ are the matrix tensile damage index and the matrix compressive damage index, respectively; when $f_{mt}\geq 1$, matrix tensile failure occurs, and when $f_{mc}\geq 1$, matrix compressive failure occurs.

Following the prediction of a particular failure mode using the above criteria, an appropriate damage variable controlled the post-failure response. The damage variables used in this model included the fiber damage variable $d_{f}$ and the matrix damage variable $d_{m}$. The fiber damage variable $d_{f}$ includes the fiber tensile damage variable $d_{ft}$ and the fiber compression damage variable $d_{fc}$. Similarly, the matrix damage variable $d_{m}$ includes the matrix tensile damage variable $d_{mt}$ and the matrix compression damage variable $d_{mc}$. The damage variable ranges from 0 to 1. When no damage occurs, the value is 0, and the value of 1 indicates the composite laminates are completely degraded [48]. The update method of damage variables adopts the form proposed by Donadon et al. [49]:

(15) $$d^{t+\Delta t}=\max\left\{0,d^{t},min\left\{1,1-\frac{\varepsilon^{0}}{\varepsilon }\left[1+\kappa^{2}\left(2\kappa -3\right)\right]\right\}\right\},$$

(16) $$\;\kappa =\frac{\varepsilon -\varepsilon^{0}}{\varepsilon^{f}-\varepsilon^{0}},$$

where $d^{t+\Delta t}$ is the damage variable of the current incremental step, $d^{t}$ is the damage variable of the previous incremental step, ε is the equivalent strain of the current incremental step, $\varepsilon^{0}$ is the equivalent strain at the beginning of damage, and $\varepsilon^{f}$ is the equivalent strain at the final failure.

**Figure 6:**
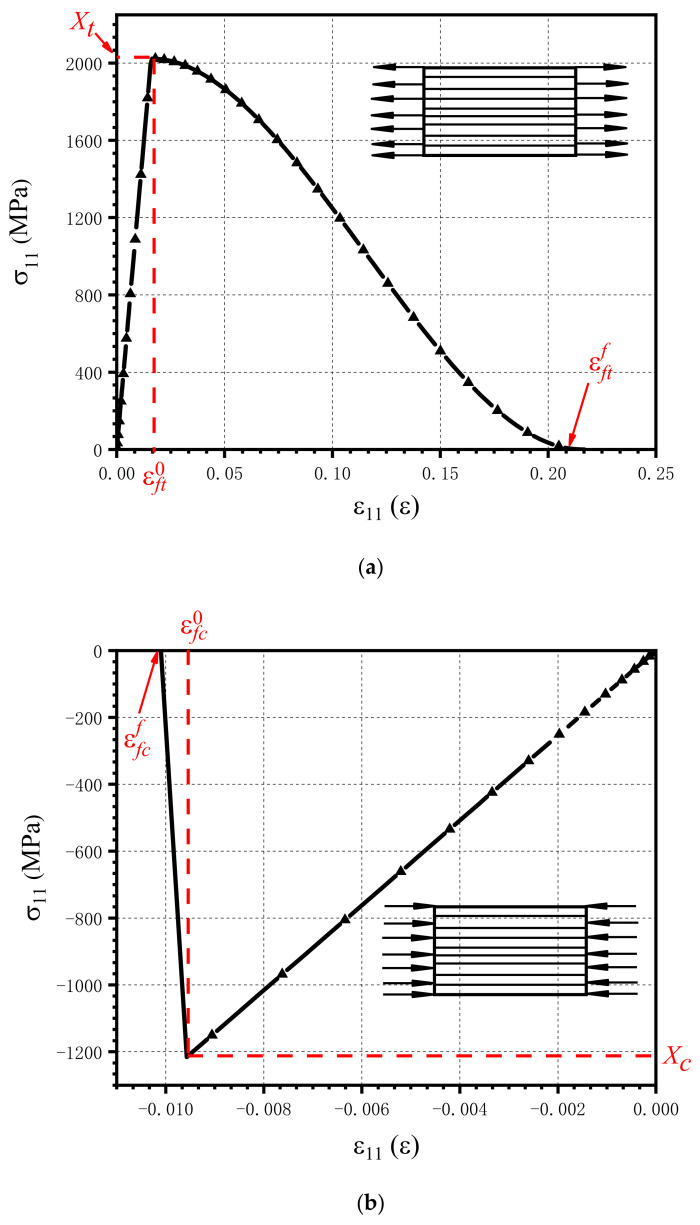
An illustration of the equivalent strain at the onset of damage and at the end of failure in the fiber direction: (**a**) the equivalent strain under tensile test and (**b**) the equivalent strain under compression test.

For fiber damage, the equivalent strain of the current incremental step is the strain component in the fiber direction. The calculations of the equivalent strain at the onset of damage and at the end of failure are as follows:

(17) $$\varepsilon_{ft}^{0}=\frac{X_{T}}{E_{1}},$$

(18) $$\varepsilon_{fc}^{0}=\frac{X_{C}}{E_{1}},$$

(19) $$\varepsilon_{ft}^{f}=\frac{2G_{ft}}{X_{T}L}\left(f_{ft}\geq 1\right),$$

(20) $$\varepsilon_{fc}^{f}=\frac{2G_{fc}}{X_{C}L}\left(f_{fc}\geq 1\right),$$

where $G_{ft}$ and $G_{fc}$ are the fracture energies of fiber tensile failure and fiber compressive failure, respectively, and L is the characteristic element length. These four equivalent strains are illustrated in Figure 6. Because there is no plastic deformation along the fiber direction, the parameters in Equations (17)–(20) do not vary with the progression of plastic deformation and damage. Under tensile load, stress softening is nonlinear, but it is linear under compression load. This is due to the fact that the equivalent strain at the onset of the damage is close to that at the end of the failure.

For the damage to the matrix, when either Equation (12) or Equation (13) is satisfied, the matrix is damaged and the equivalent stress and the equivalent strain are formulated as:

(21) $$\sigma_{m}^{0}={\left.\sqrt{\sigma_{n}{}^{2}+{\left(\sigma_{nL}\right)}^{2}+{\left(\sigma_{nT}\right)}^{2}}\right|}_{d_{m}^{t+\Delta t}=0},$$

(22) $$\varepsilon_{m}^{0}={\left.\sqrt{\varepsilon_{n}{}^{2}+{\left(\varepsilon_{nL}\right)}^{2}+{\left(\varepsilon_{nT}\right)}^{2}}\right|}_{d_{m}^{t+\Delta t}=0},$$

where, $\varepsilon_{n}$, $\varepsilon_{nL}$, and $\varepsilon_{nT}$ are the normal traction and shear tractions on the fracture surface, respectively; $d_{m}^{t+\Delta t}$ is the matrix damage variable of the current incremental step; and x is the Macaulay bracket, defined as $\langle x\rangle =\max\left(0,x\right)$. After the damage, the equivalent strain of the matrix in the current incremental step becomes

(23) $$\varepsilon_{m}={\left.\sqrt{\varepsilon_{n}{}^{2}+{\left(\varepsilon_{nL}\right)}^{2}+{\left(\varepsilon_{nT}\right)}^{2}}\right|}_{d_{m}^{t+\Delta t}>0}\;\left(f_{mt}\geq 1\right),$$

(24) $$\varepsilon_{m}={\left.\sqrt{{\left(\varepsilon_{nL}\right)}^{2}+{\left(\varepsilon_{nT}\right)}^{2}}\right|}_{d_{m}^{t+\Delta t}>0}\;\left(f_{mc}\geq 1\right),$$

For any mixed-modes failure scenario, the fracture energy for matrix failure is:

(25) $$G_{m}=G_{Ic}{\langle \frac{\sigma_{n}^{d}}{\sigma_{m}^{o}}\rangle }^{2}+G_{IIc}{\left(\frac{\sigma_{nL}^{d}}{\sigma_{m}^{o}}\right)}^{2}+G_{IIc}{\left(\frac{\sigma_{nT}^{d}}{\sigma_{m}^{o}}\right)}^{2},$$

where $G_{Ic}$ and $G_{IIc}$ are fracture energies for mode I and mode II fracture, respectively, and $\sigma_{n}^{d}$, $\sigma_{nL}^{d}$, and $\sigma_{nT}^{d}$ are the tractions at the onset of matrix failure. The final failure strain of the matrix is defined as:

(26) $$\varepsilon_{m}^{f}=\frac{2G_{m}}{\sigma_{m}^{0}L}\;.$$

When matrix damage occurs in laminates, the stress tensor is rotated to the fracture plane by Equation (11) and the relevant tractions are softened according to Equation (27), yielding $\sigma_{n}^{\prime }$, $\sigma_{nL}^{\prime }$, and $\sigma_{nT}^{\prime }$. To consider the effect of reverse load on crack closure, the normal traction is softened only when it is tensile.

(27) $$\left\{\begin{array}{c} \sigma_{n}^{\prime }=\sigma_{n}{\left.\left(1-d_{m}\right)\right|}_{\sigma_{n}>0}\\ \sigma_{nL}^{\prime }=\sigma_{nL}\left(1-d_{m}\right)\\ \sigma_{nT}^{\prime }=\sigma_{nT}\left(1-d_{m}\right) \end{array}\right.,$$

After that, the tractions on the fracture surface are converted into stress in the global coordinate system through the inverse transformation of Equation (11), yielding $\sigma_{ij}^{\prime }\left(i,j=1,2,3\right)$.

Matzenmiller et al. [50] assumed that fiber rupture damage, buckling, and compressive failure of fiber cause the damage evolution in the resin matrix. Based on this assumption, all stress components are softened:

(28) $$\left[\begin{array}{c} \sigma_{11}^{\prime \prime }\\ \sigma_{22}^{\prime \prime }\\ \sigma_{33}^{\prime \prime }\\ \sigma_{12}^{\prime \prime }\\ \sigma_{23}^{\prime \prime }\\ \sigma_{13}^{\prime \prime } \end{array}\right]=\left(1-d_{f}\right)\left[\begin{array}{c} \sigma_{11}^{\prime }\\ \sigma_{22}^{\prime }\\ \sigma_{33}^{\prime }\\ \sigma_{12}^{\prime }\\ \sigma_{23}^{\prime }\\ \sigma_{13}^{\prime } \end{array}\right],$$

where $\sigma_{ij}^{\prime \prime }\left(i,j=1,2,3\right)$ is the softened stress after fiber failure.

### 3.2. Calculation Procedure

**Figure 7:**
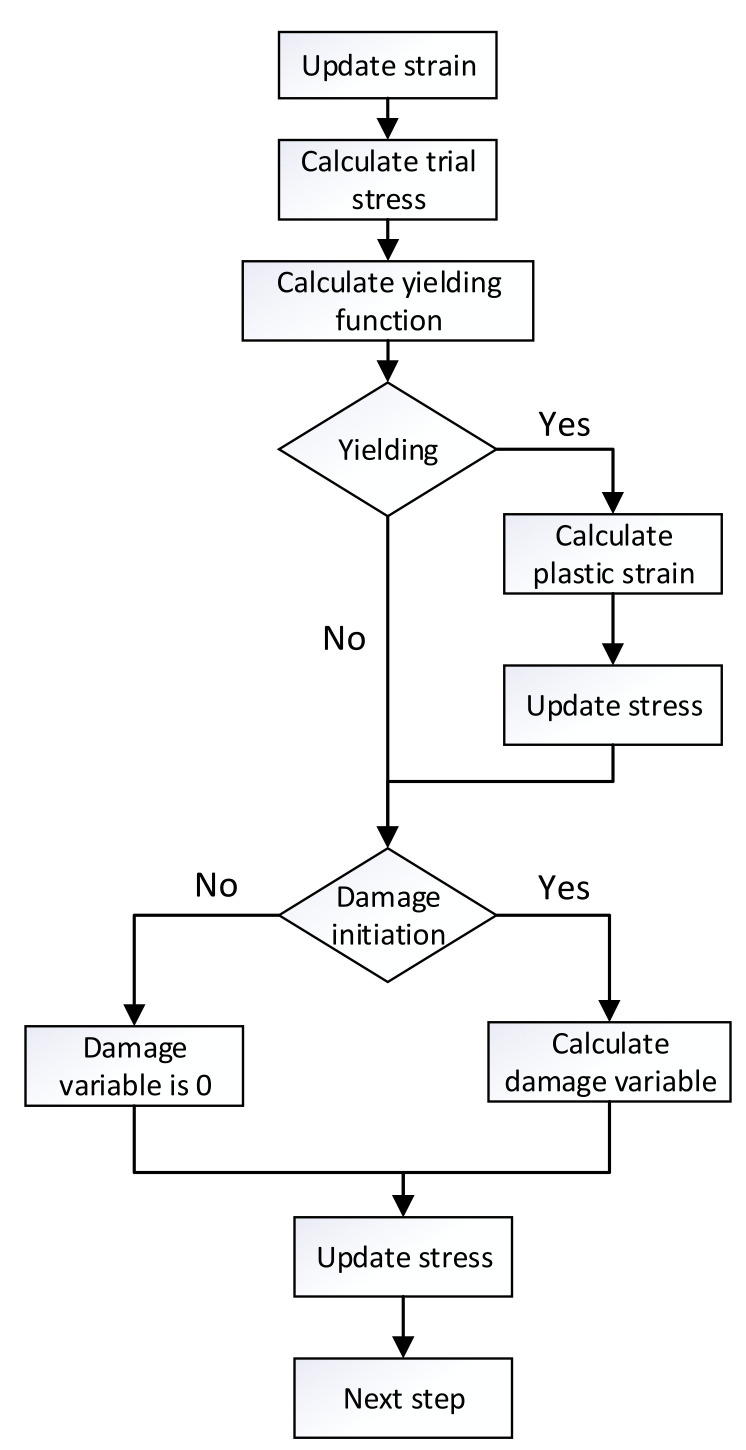
Calculation process of the 3D elastoplastic damage model.

According to the numerical algorithm of the 3D elastoplastic damage model, a user-defined material subroutine VUMAT based on ABAQUS was compiled. A calculation flow chart is shown in Figure 7. Ren et al. [51] proved that the forward Euler method and the backward Euler method can accurately solve the plastic strain. Considering that the algorithm used in this paper is an explicit dynamic algorithm and the stable time increment is $10^{-8}\;$ s, the forward Euler algorithm [52] was used to solve the plastic strain to minimize the computation time. The numerical simulation was carried out by step-by-step displacement loading. The calculation procedure in the *n* + 1 step was as follows:

(1) Initial conditions:

$$\Delta \varepsilon_{n+1},\varepsilon_{n},\varepsilon_{n}^{p},\bar{\varepsilon_{n}^{p}},$$

where $\Delta \varepsilon_{n+1}$ is the strain tensor increment in step *n* + 1 and $\varepsilon_{n}$, $\varepsilon_{n}^{p}$, and $\bar{\varepsilon_{n}^{p}}$ are the total strain tensor, the plastic strain tensor, and the equivalent plastic strain, respectively, in step *n*.

(2) Assuming that the plastic strain tensor and the equivalent plastic strain of the current step are consistent with those of the previous step, the trial stress in the current step is calculated by:

$$\varepsilon_{n+1}^{p,trial}=\varepsilon_{n}^{p},\bar{\varepsilon_{n+1}^{p,trial}}=\bar{\varepsilon_{n}^{p}},\varepsilon_{n+1}=\varepsilon_{n}+\Delta \varepsilon_{n+1},\sigma_{n+1}^{trial}=C:\left(\varepsilon_{n+1}-\varepsilon_{n+1}^{p,trial}\right),$$

where $\varepsilon_{n+1}^{p,trial}$ and $\bar{\varepsilon_{n+1}^{p,trial}}$ are the trial plastic strain tensor and the trial equivalent plastic strain, respectively, in step *n* + 1; $\varepsilon_{n+1}$ is the strain tensor in step *n* + 1; and $\sigma_{n+1}^{trial}$ is the trial stress tensor in step *n* + 1.

(3) Check the yield criterion:

$$F_{n+1}\left(\sigma_{n+1}^{trial},\bar{\varepsilon_{n+1}^{p,trial}}\right)=F^{p}\left(\sigma_{n+1}^{trial}\right)-\kappa \left(\bar{\varepsilon_{n+1}^{p,trial}}\right).$$

If $F_{n+1}\left(\sigma_{n+1}^{trial},\bar{\varepsilon_{n+1}^{p,trial}}\right)\leq 0$, then the plastic strain tensor $\varepsilon_{n+1}^{p}$, the equivalent strain $\bar{\varepsilon_{n+1}^{p}}$ and the stress tensor $\sigma_{n+1}$ in step *n* + 1 are $\varepsilon_{n+1}^{p}=\varepsilon_{n}^{p}$, $\bar{\varepsilon_{n+1}^{p}}=\bar{\varepsilon_{n}^{p}}$, $\sigma_{n+1}=\sigma_{n+1}^{trial}$, respectively; otherwise, go to Equation (4) to calculate plastic strain increments.

(4) Calculate the plastic strain and the update stress:
  (a) Calculate the scale factor increment:
    $$\Delta \lambda_{n+1}=\frac{\frac{\partial F_{n+1}\left(\sigma_{n+1}^{trial},\bar{\varepsilon_{n+1}^{p,trial}}\right)}{\partial \sigma_{n+1}^{trial}}:C:\Delta \varepsilon_{n+1}}{\frac{\partial F_{n+1}\left(\sigma_{n+1}^{trial},\bar{\varepsilon_{n+1}^{p,trial}}\right)}{\partial \sigma_{n+1}^{trial}}:C:\frac{\partial F_{n+1}\left(\sigma_{n+1}^{trial},\bar{\varepsilon_{n+1}^{p,trial}}\right)}{\partial \sigma_{n+1}^{trial}}-\frac{\partial F_{n+1}\left(\sigma_{n+1}^{trial},\bar{\varepsilon_{n+1}^{p,trial}}\right)}{\partial \bar{\varepsilon_{n+1}^{p,trial}}}}.$$
  (b) Update the plastic strain tensor and the equivalent plastic strain:
    $$\varepsilon_{n+1}^{p}=\varepsilon_{n}^{p}+\Delta \lambda_{n+1}\frac{\partial F_{n+1}\left(\sigma_{n+1}^{trial},\bar{\varepsilon_{n+1}^{p,trial}}\right)}{\partial \sigma_{n+1}^{trial}},\bar{\varepsilon_{n+1}^{p}}=\bar{\varepsilon_{n}^{p}}+\Delta \lambda_{n+1}.$$
  (c) Update the stress tensor:

$$\sigma_{n+1}=C:\left(\varepsilon_{n+1}-\varepsilon_{n+1}^{p}\right).$$

(5) Judge whether damage has been initiated and calculate the damage variable.
  (a) Judge the positive and negative values of $\sigma_{11}$, and judge whether fiber damage has been initiated according to different fiber damage initiation criteria. If fiber damage occurs, the corresponding fiber damage variable $d_{f}$ is calculated. Otherwise, $d_{f}=0$.
  (b) The stress is rotated to the fracture surface to determine whether the tractions on the fracture surface satisfy the damage initial criteria of the matrix. If matrix damage occurs, the corresponding matrix damage variable $d_{m}$ is calculated. Otherwise, $d_{m}=0$.

(6) Update the stress according to the calculated fiber damage variable and the matrix damage variable and skip to the next step.

### 3.3. Finite Element Model

The material properties, failure strength, and fracture energy of AS4/PEEK are shown in Table 2 and Table 3 [45,51,53,54]. These material parameters were determined by a series of mechanical tests, including tensile tests, compression tests, off-axis tensile tests, and double cantilever beam tests. The elastic modulus and the Poisson ratio of the countersunk bolt were 110,000 MPa and 0.3, respectively, and the plastic deformation of the countersunk bolt was not considered in this paper.

**Table 2:** Material properties of AS4/PEEK.

$E_{1}\;\left(MPa\right)$	$E_{2}\;\left(MPa\right)$	$E_{3}\;\left(MPa\right)$	$G_{12}\;\left(MPa\right)$	$G_{13}\;\left(MPa\right)$	$G_{23}\;\left(MPa\right)$
127,000	10,300	10,300	6000	6000	3450
$\upsilon_{12}$	$\upsilon_{13}$	$\upsilon_{23}$	$a_{66}$	β	n
0.32	0.32	0.49	1.50	292.67	0.135

**Figure 8:**
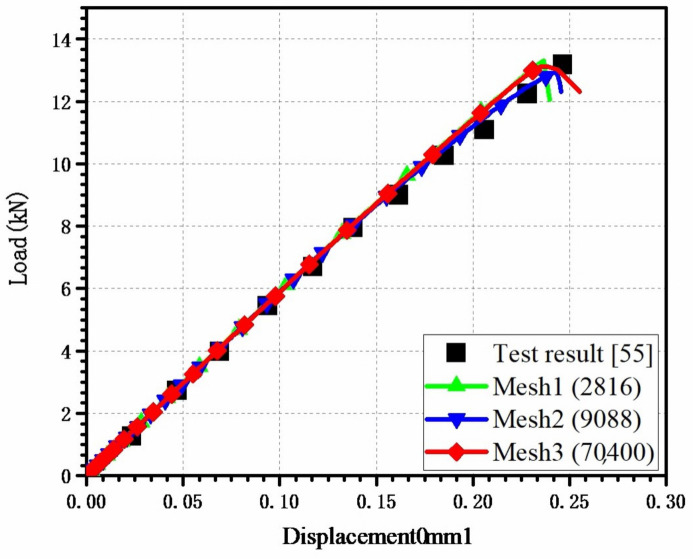
Comparison of load–displacement curves with different mesh densities.

The numerical simulation of tensile tests of AS4/PEEK laminates with holes was performed to investigate the effect of the mesh size on the 3D elastoplastic model. The test results data were obtained from Ref. [55], and there were three models in all. All models used linear, eight-node, three-dimensional, reduced-integration (C3D8R) element, and the total number of meshes were 2816, 9088, and 70,400, respectively. The model’s dimension and boundary conditions were consistent with those of the test. As illustrated in Figure 8, the load–displacement curves acquired by the numerical simulation were compared to those obtained through testing. The stiffness and strength of the three models were close to the experimental values, suggesting that the 3D elastoplastic model is less dependent on mesh size. The above conclusions remained unchanged as the mesh was refined, which indicates that the influence of mesh size on calculation results can be ignored within a reasonable range of mesh size.

**Figure 9:**
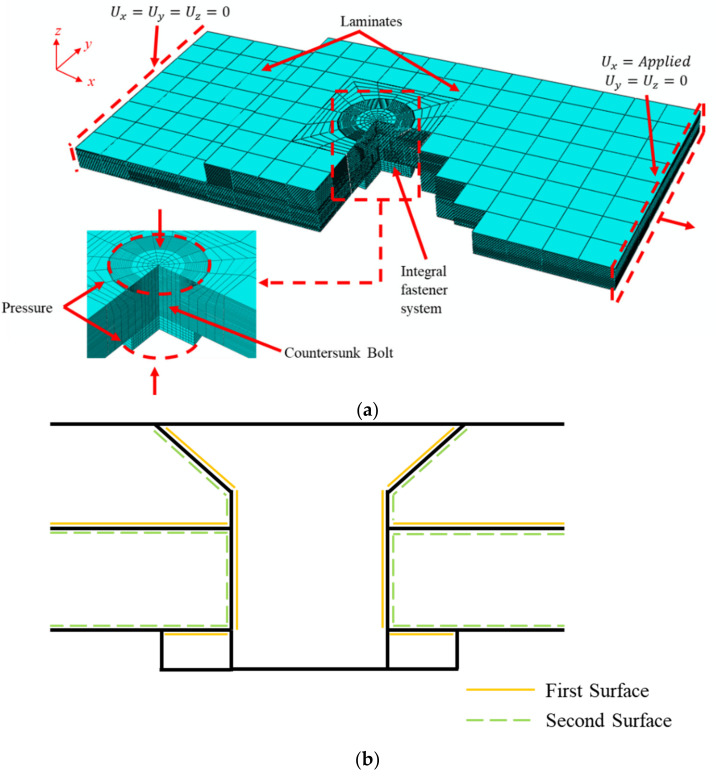
(**a**) Finite element model of single-lap thermoplastic composite joints bolted by countersunk with boundary conditions. (**b**) Surface definition for contact pairs around the countersunk bolt.

To reduce calculation time, the finite element model of the countersunk bolted joint did not consider the pads of the test specimens. We also ignored the effect of the screw thread. Thus, the countersunk bolt and nut were built as a whole model. The simplified finite element model is shown in Figure 9a, where the x–y–z coordinate system is the global coordinate system and the x-direction is parallel to the fiber direction. The analysis procedure was divided into two steps, where the first step implemented the tightening torque applied to the bolt. The tightening torques were 1.8 N·m, 3.6 N·m, 5.4 N·m, and 1.8 N·m, corresponding to the tightening torque of the test specimens. The bolt-tightening torque was simulated by applying an equivalent pressure load on both sides of the bolt. The normal pressure ($p_{w}$) acting on the bolt surface was by the equation $p_{w}=T/\left(\Lambda \times D\times A\right)$, where T is the applied bolt torque; Λ is the torque coefficient, which is commonly assumed to be 0.2 [56]; and D and A are the diameter and the area, respectively, of the upper and lower surfaces of the countersunk bolt. In the second step, the left end of the model was held fixed, while a 5 mm displacement with a smooth step amplitude curve along the *x*-displacements was applied to the right hand. The step time was by $t=10/f^{1st}$, where $f^{1st}$ is the fundamental frequency of the laminate [57]. The frequency analysis showed that the laminates had a fundamental frequency of 1116 Hz, and it was appropriate to set the time step to 0.01 s in both steps.

The contact properties included the normal behavior and the tangential behavior; the normal behavior uses hard contact, whereas the friction formulation in the tangential behavior uses the penalty formulation. Contact surfaces included multiple assembly-level interactions that used the friction coefficient of laminate–laminate interaction (μ=0.7) and bolt–laminate interaction (μ=0.1) [58]. The setting of the contact pair is shown in Figure 9b. The first surface and the second surface were the contact surfaces used in the general contact. The general contact algorithm of ABAQUS/Explicit uses a finite-sliding formulation and enforced-penalty-based, stick–slip interactions. The bolt-tightening torque was simulated by applying an equivalent pressure load on both sides of the bolt. In the model, the clearances between the laminate and the bolt were 0 μm, 60 μm, 120 μm, and 180 μm, which are 0%, 1%, 2%, and 3% of the bolt diameter, respectively, and 0 μm corresponds to the clearance of the test specimens. The above models with different clearances were named C0, C1, C2, and C3, respectively. The computation accuracy was set as “double precession” to reduce the accumulation of errors when running the simulation. The mesh type was C3D8R for the bolt and laminates, and each layer was separated into its elements in the through-thickness direction to get the stress distribution of and damage to each layer. At the same time, to improve the calculation accuracy of local stress, the mesh was refined at the hole edge. The mesh size of the bolt surface was consistent with that of the hole edge of the laminate in order to make the mesh of the bolt correspond to that of the hole edge of the laminate. The total mesh number was 23,340.

## 4. Numerical Simulation Results

### 4.1. Model Verification

**Figure 10:**
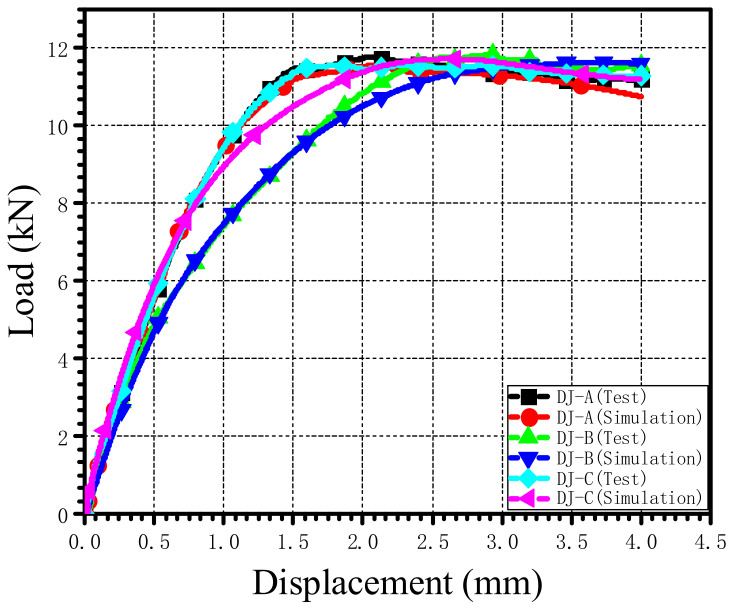
Comparison of load–displacement curves between numerical simulation and the test.

A comparison of load–displacement curves of the single-lap countersunk bolted joints with three different stacking sequences obtained from simulation and tests is shown in Figure 10. It can be seen that the load–displacement curves obtained by numerical simulation were in good agreement with those from the tests, which indicates that the model is suitable for simulating mechanical behaviors of AS4/PEEK laminate single-lap countersunk bolted joints under tensile load.

A comparison of the ultimate bearing strength and the 2% offset bearing strength obtained by numerical simulation and test is summarized in Table 4. The maximum error of the ultimate bearing strength and the 2% offset bearing strength was 5.23%, indicating that the model can accurately calculate the ultimate bearing strength and the 2% offset bearing strength of single-lap countersunk bolted joints of AS4/PEEK laminates under tensile load.

**Figure 11:**
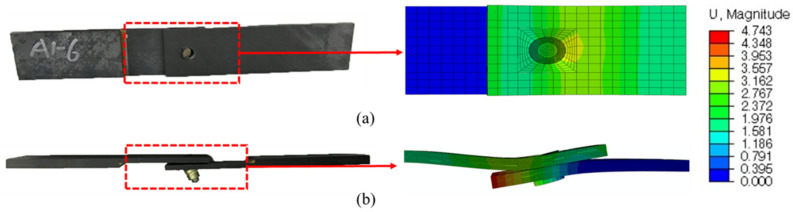
Comparison of displacement between the test specimen and numerical simulation: (**a**) nail-head side and (**b**) lateral side.

**Figure 12:**
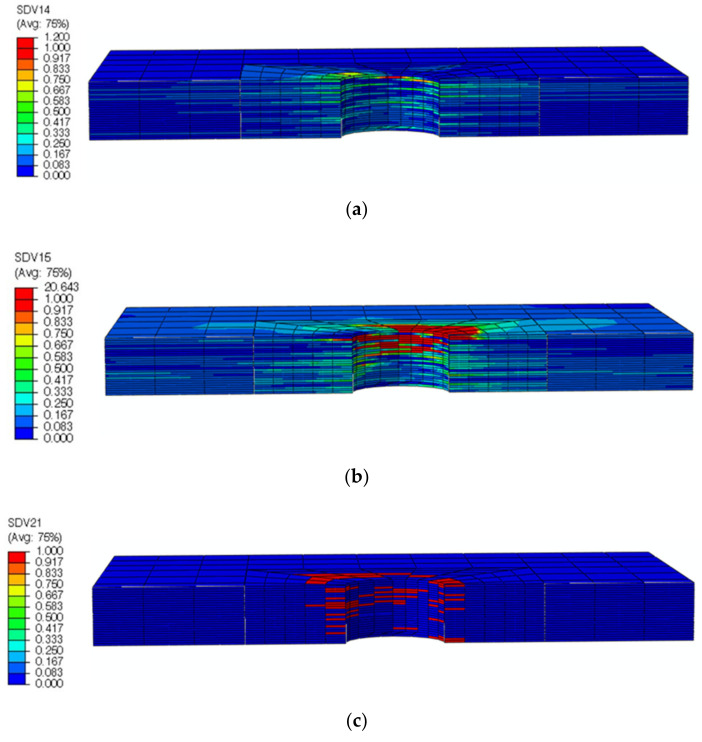

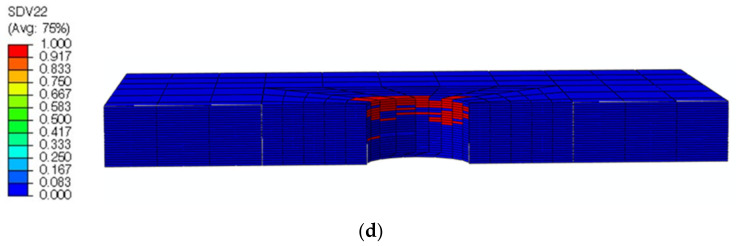
Damage index of the lower laminate around the hole. Damage occurs when the damage index is greater than or equal to 1. (**a**) Fiber tensile damage index, (**b**) fiber compressive damage index, (**c**) matrix tensile damage index, and (**d**) matrix compressive damage index.

**Figure 13:**
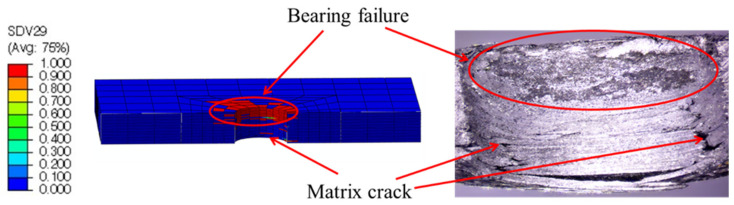
Comparison of damage modes between numerical simulation and the test.

Taking the DJ-A model as an example, the displacements obtained from the numerical simulation and the specimen after the test are shown in Figure 11. The bending moment generated by the eccentric load path caused the laminates to bend, which was well simulated by the numerical simulation. As shown in the numerical simulation results, the highest displacement occurred near the left end of the lower laminate, which is consistent with the test results. The damage index and the damage variable are shown in Figure 12 and Figure 13, respectively, where SDV29 is the matrix damage variable. The comparison in Figure 13 shows that the numerical simulation was accurate in simulating the damage to the countersunk bolted joint. The damage in the laminates was mainly matrix damage, and the matrix compressive damage mainly occurred in the upper part of the hole edge of the laminates, while the matrix tensile damage was distributed in the upper and lower parts of the hole edge. The fiber damage mainly occurred in the upper part of the hole edge of the laminates and was mainly fiber compressive damage.

**Figure 14:**
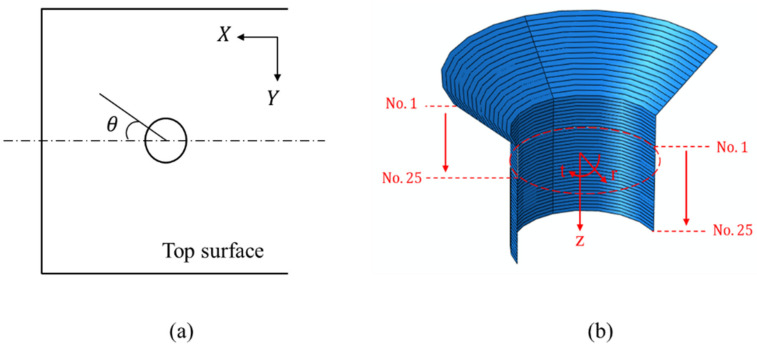
Definition of a stress field around the bolt hole: (**a**) bolt–hole contact angle definition and (**b**) ply number definition.

**Figure 15:**
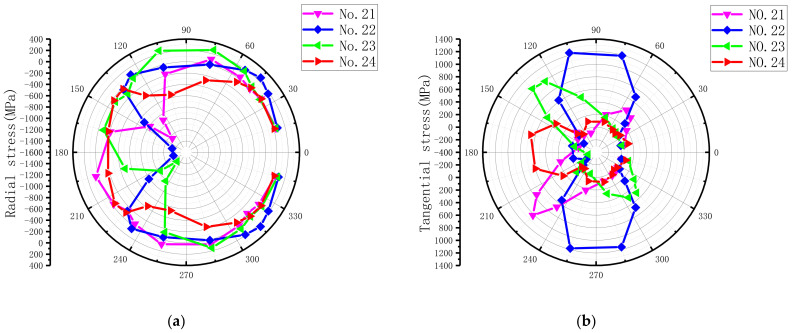

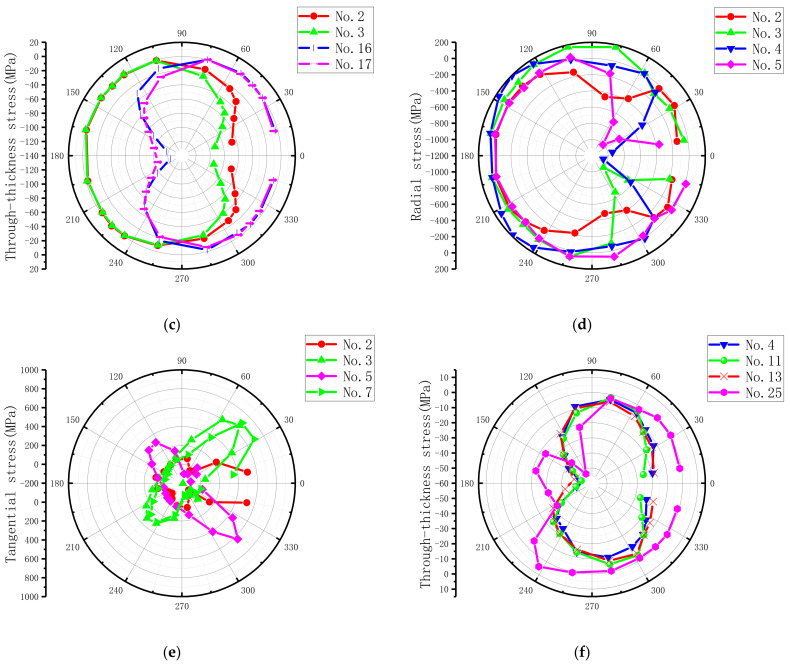
Stress distribution of the DJ-A model under 6 kN external load. (**a**) Radial stress in the upper plate, (**b**) tangential stress in upper laminates, (**c**) through-thickness stress in upper laminates, (**d**) radial stress in lower laminates, (**e**) tangential stress in lower laminates, and (**f**) through-thickness stress in lower laminates.

For ease of description, as illustrated in Figure 14, a polar coordinate system and the bolt–hole contact angle (θ) were defined to present the countersunk hole boundary stresses, which included the radial (r) stress, the tangential (t) stress, and the through-thickness (z) stress. The laminate plies were numbered 1–25, beginning at the top surface. The stresses were extracted from the integration points closest to the hole edge at an applied load level of 6 kN. The load level belonged to the elastoplastic region to ensure the hole was undamaged. The radial peak stress, the tangential peak stress, the through-thickness peak stress, and the ply number at different ply angles are summarized in Table 5, and the stress distribution of the corresponding layers is plotted in Figure 15. The peak values of radial stress and tangential stress mainly appeared in the 0° layer, while the peak values of radial stress and tangential stress in the 90° layer were the smallest. Therefore, we can conclude that the external load is mainly borne by the 0° layer, followed by the ±45° layer, and the load borne by the 90° layer is the smallest. 

From the stress distribution in Figure 15, it can be clearly seen that the peak value of radial stress appeared in the position parallel to the fiber direction and was dominated by compressive stress. The radial stress distribution range around the hole circumference, which represents the bolt–hole contact arc, was approximately a semicircle. This was due to the absence of the bolt–hole clearance (0 μm) in this model. The peak tangential stress emerged at the location tangent to the fiber direction, which is due to the plies being the stiffest at these locations [13]. The peak tangential stress was predominantly tensile stress, but tangential compressive stresses existed at some sites owing to hole oval deformation, as found by Ref. [13]. The radial peak stress and the tangential peak stress were mainly distributed in the middle part of the countersunk bolted joint (i.e., the lower part of the upper plate and the upper part of the lower plate), which is in agreement with the results of Egan et al. [59] and Zhai et al. [60]. For the peak value of through-thickness stress, its value was only about 5–15% of the peak values of radial stress and tangential stress, and it was mainly compressive stress. This is because the through-thickness stress is mainly generated by the bolt-tightening torque. 

### 4.2. Influence of Bolt-Tightening Torque

**Figure 16:**
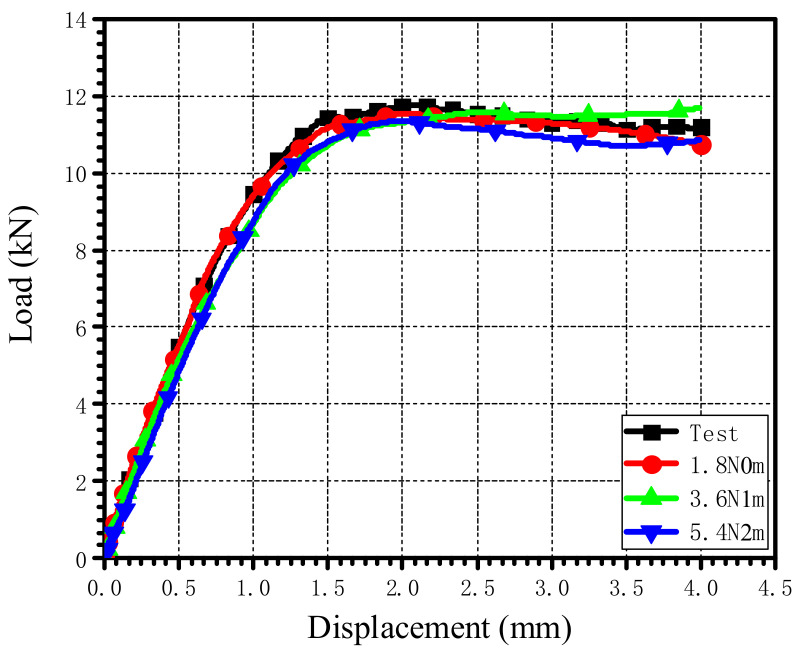
Load-displacement curves under different bolt-tightening torques when the bolt-hole clearance is 0 μm.

**Figure 17:**
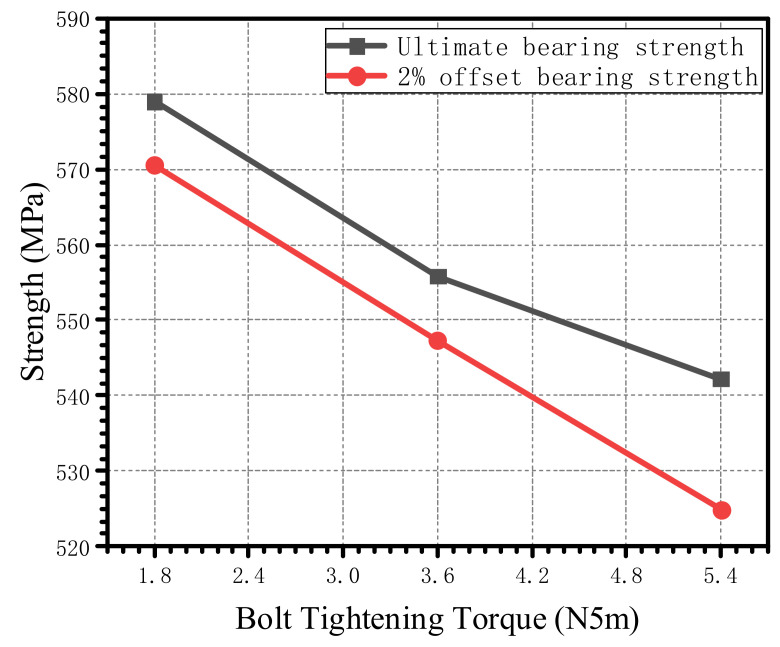
Ultimate bearing strength and 2% offset bearing strength under different bolt-tightening torques when the bolt–hole clearance is 0 μm.

By changing the bolt-tightening torque applied on the bolt, the load–displacement curve obtained by a numerical simulation is shown in Figure 16. The ultimate bearing strength and the 2% offset bearing strength obtained by numerical simulations under different tightening torques are plotted in Figure 17. When the bolt-tightening torque increased from 1.8 N·m to 5.4 N·m, the ultimate bearing strength decreased by 6.34%, while the 2% offset bearing strength decreased by 8.01%.

**Figure 18:**
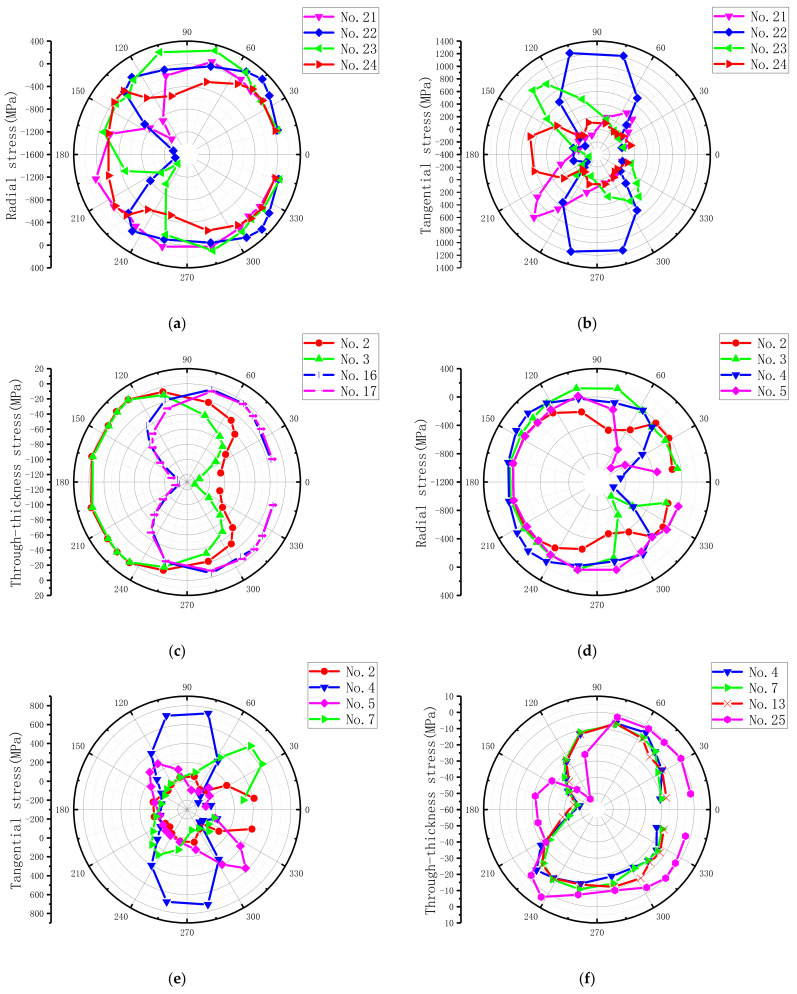
Stress distribution when the external load is 6 kN, the clearance remains 0 μm, and the bolt-tightening torque is 5.4 N·m. (**a**) Radial stress in the upper plate, (**b**) tangential stress in upper laminates, (**c**) through-thickness stress in upper laminates, (**d**) radial stress in lower laminates, (**e**) tangential stress in lower laminates, and (**f**) through-thickness stress in lower laminates.

The integral point stress on the laminate near the countersunk bolt was extracted when the bolt-tightening torque was 5.4 N·m and the external load was 6 kN. The peak stress at different ply angles and the layer number obtained by a numerical simulation are summarized in Table 6, and the stress distribution of the above layers is plotted in Figure 18. Compared to Figure 15, it can be clearly seen that increasing the external load did not change the stress distribution trend but changed the peak stress. The difference in peak stresses is shown in Table 7. It shows that when the bolt-tightening torque increased from 1.8 N·m to 5.4 N·m, the peak values of radial stress and tangential stress in the upper plate changed little, while in the lower plate, they changed greatly; the peak values decreased, and the maximum decline reached 18.92%. For the through-thickness peak stress, the upper laminate increased except for 0° ply, and the maximum increase reached 27.70%, while in the lower laminate, it decreased, and the maximum decrease reached 10.42%. Therefore, increasing the bolt-tightening torque within a certain range can effectively reduce the peak radial stress and the peak tangential stress. However, the increased through-thickness stress leads to laminate damage under low external load, resulting in a slight decrease in the ultimate bearing strength and the 2% offset bearing strength.

### 4.3. Influence of Clearance

**Figure 19:**
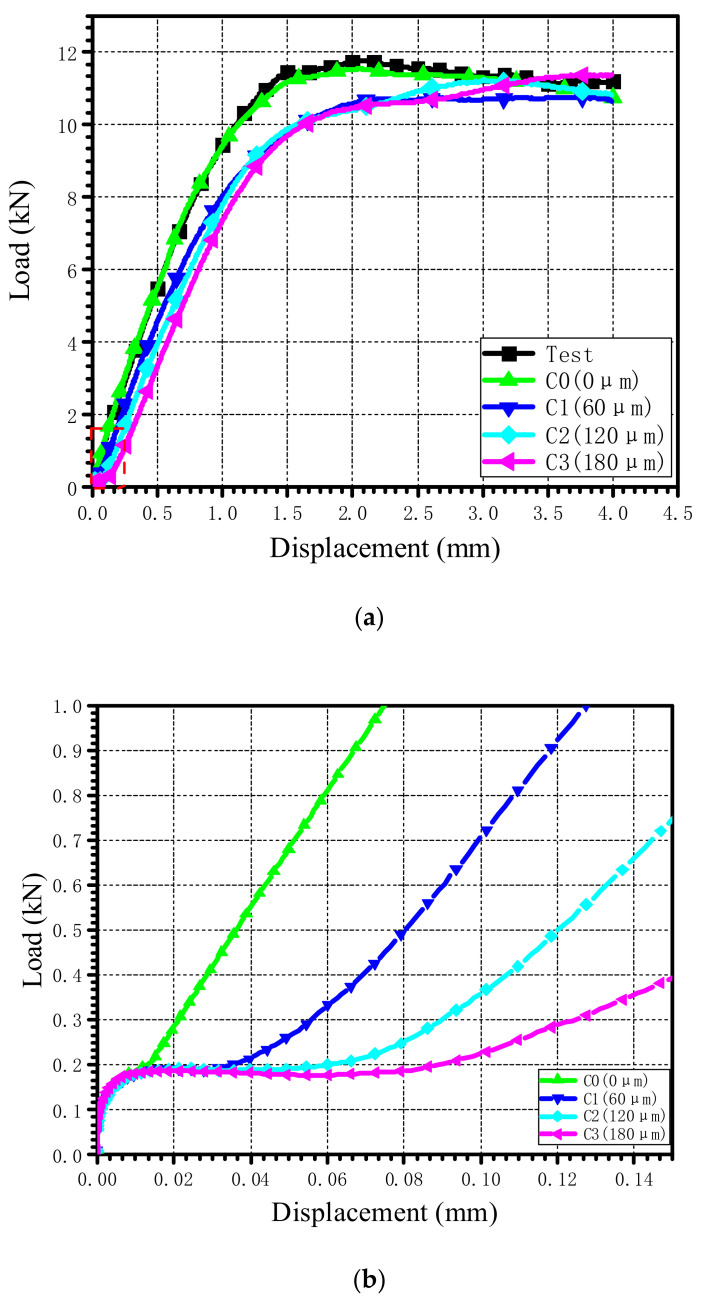
(**a**) Load–displacement curves under different clearances. (**b**) Local amplification of load–displacement curves under different clearances.

**Figure 20:**
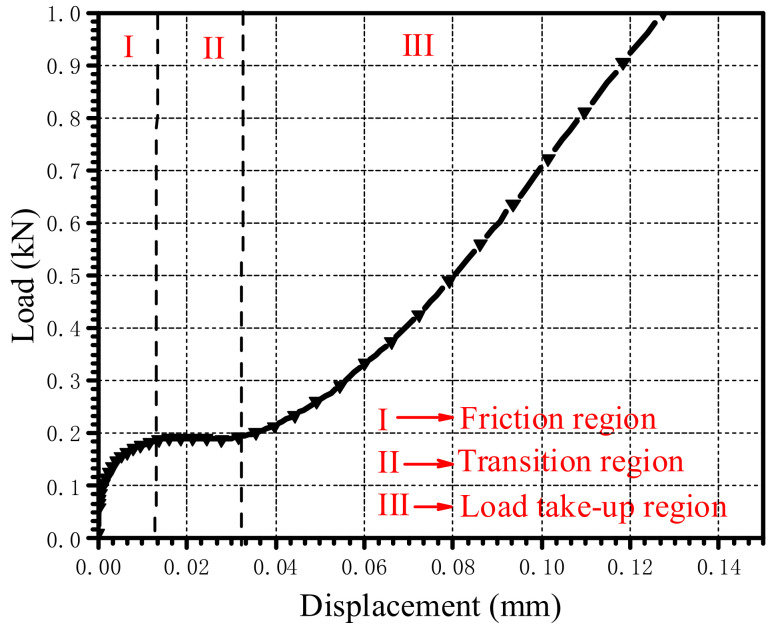
Three regions of the load–displacement curve.

**Figure 21:**
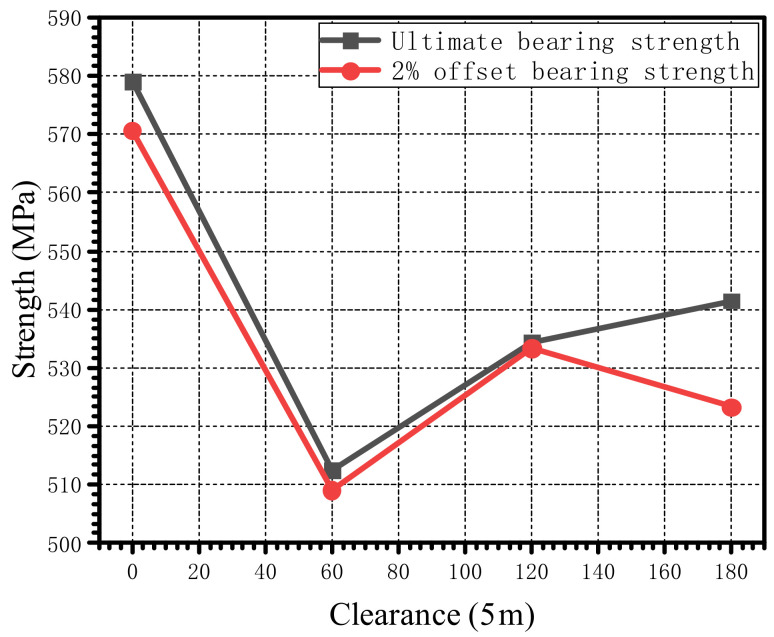
Ultimate bearing strength and 2% offset bearing strength under different clearances.

The load–displacement curves under different clearances are shown in Figure 19a. To study the influence of the clearance on the countersunk bolted joint when the displacement is small, the load–displacement curve when the displacement is less than 0.14 mm is plotted in Figure 19b. As shown in Figure 20, for the joint with clearances, it is obvious that there were three regions: the friction region, the transition region, and the load take-up region. The friction region was mainly produced by the friction force between the laminates; when the external load increased to a certain value, the upper and lower laminated plates began to slide rigidly, the slope was small, and the rigid displacement distance was about half of the clearance length. After the transition region, the laminates and the countersunk bolts were in complete contact, and the load began to increase gradually. The ultimate bearing strength and the 2% offset bearing strength corresponding to different clearances are plotted in Figure 21. When the clearance increased from 0 μm to 60 μm, the decrease in the ultimate bearing strength and the 2% offset bearing strength was the largest, at 11.47% and 10.78%, respectively. With the increase in the clearance, the ultimate bearing strength increased gradually and the 2% offset bearing strength increased first and then decreased.

**Figure 22:**
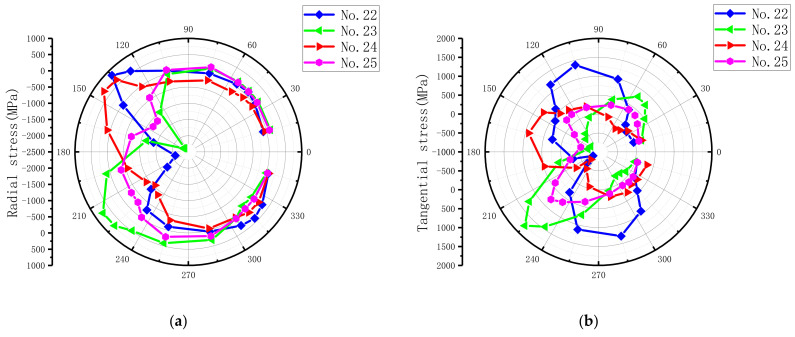

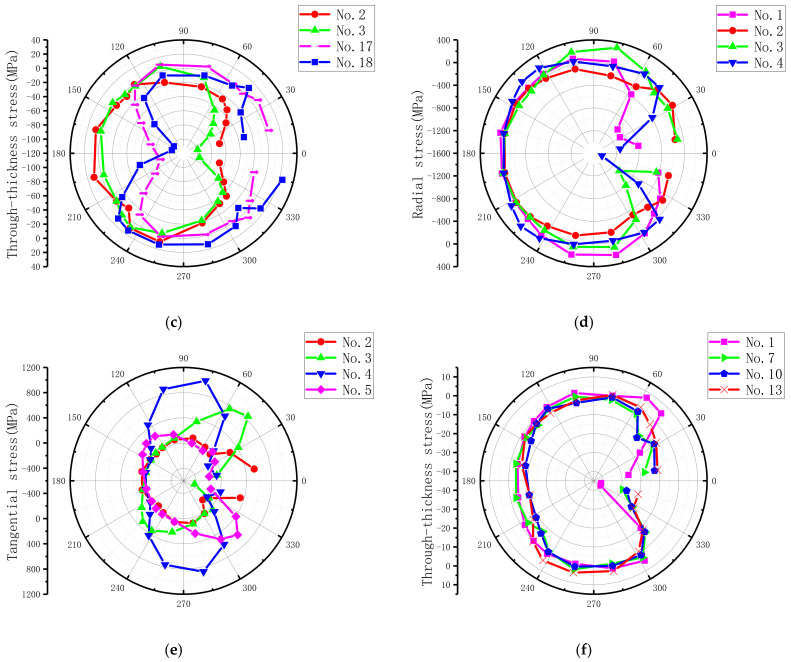
Stress distribution when the external load is 6 kN, the bolt-tightening torque remains 1.8 N·m, and the clearance is 180 μm. (**a**) Radial stress in the upper plate, (**b**) tangential stress in upper laminates, (**c**) through-thickness stress in upper laminates, (**d**) radial stress in lower laminates, (**e**) tangential stress in lower laminates, and (**f**) through-thickness stress in lower laminates.

When the clearance was 180 μm and the external load was 6 kN, the integral point stresses on the laminate near the countersunk bolt were extracted. The peak stress of different ply angles and the corresponding ply number are summarized in Table 8, and the stress distribution of the above layer is drawn in Figure 22. In comparison to Figure 15, the peak stress positions and values changed; the radial stress distribution range in the counterclockwise direction was approximately 105–255° in the upper laminate and 300–360° and 0–60° in the lower laminate; the contact area decreased by 16.7% in the upper laminate and 33.3% in the lower laminate. A decrease in the contact area led to an increase in the radial peak stress. This finding is consistent with that of Ref. [13]. The peak stress difference between the model and the model without clearance is summarized in Table 9. The peak value of radial stress increased except in the −45° layer of the upper layer and the 90° layer of the lower layer, and the maximum increase reached 90.94%. The tangential stress increased except in the −45° layer, and the maximum increase was 70.74%. For the peak stress in the through-thickness direction, except for the 45° layer of the upper laminate, the peak stress decreased and the maximum decrease was 57.33%. The decrease in the lower laminate was larger than that in the upper laminate. Therefore, the main influence of clearance on the single-lap countersunk bolted joint is to increase the peak values of radial stress and tangential stress, and it has a certain mitigation effect on the peak value of the through-thickness stress.

## 5. Conclusions

In this paper, tensile tests were performed on single-lap countersunk bolt thermoplastic joints where AS4/PEEK laminates were stacked in three sequences. The ultimate failure of all test specimens occurred in the laminates around the countersunk bolt, and the effect of stacking sequences on both ultimate bearing strength and 2% offset bearing strength was limited. Furthermore, a 3D elastoplastic damage model was established and used in the numerical simulation of single-lap countersunk bolted joints. The numerical method of stiffness estimation was validated by comparing with the test stress–strain curves. The maximum error of predicted strength was only 5.23%, which proves that the present FEM can also accurately simulate the ultimate strength of AS4/PEEK laminate joints bolted by countersunk. 

The proposed numerical model was used to analyze the effects of bolt-tightening torque and clearance on ultimate bearing strength and 2% offset bearing strength. When the bolt-tightening torque was increased from 1.8 N·m to 5.4 N·m, the ultimate bearing strength decreased by 6.34%, and the 2% offset bearing strength decreased by 8.01%. The analysis of the peak stress at different ply angles shows that increasing the bolt-tightening torque within a certain range can remarkably reduce the peak values of radial stress and tangential stress and the decrease in ultimate bearing strength and 2% offset bearing strength is mainly due to the increase in the through-thickness peak stress. When the clearance was increased from 0 μm to 60 μm with a constant bolt-tightening torque of 1.8 N·m, the ultimate bearing strength and the 2% offset bearing strength fell by 11.47% and 10.78%, to a minimum value, respectively. However, when the clearance was enlarged, the trend of ultimate bearing strength and 2% offset bearing strength was different: the ultimate bearing strength increased steadily, but the 2% offset bearing strength increased first and then decreased. In addition, the numerical results show that the size of the clearance will affect the value and position of peak radial and tangential stresses, which is mainly due to changes in the contact position and the contact area.

## Figures and Tables

**Table 1:** The bearing strength of AS4/PEEK single-lap countersunk bolted joints with three stacking sequences.

	Ultimate Bearing Strength (MPa)	2% Offset Bearing Strength (MPa)
DJ-A	560.50	542.20
DJ-B	562.36	534.33
DJ-C	564.40	561.40

**Table 3:** Strength and fracture energy of AS4/PEEK.

$X_{T}\;\left(MPa\right)$	$X_{C}\;(\mathrm{MPa})$	$Y_{T}\;\left(MPa\right)$	$Y_{c}\;\left(MPa\right)$	$S_{L}\;\left(MPa\right)$	$G_{Ic}\;\left(kJ\cdot m^{-2}\right)$
2023	1234	92.7	176	82.6	1.7
$G_{IIc}\left(\mathrm{kJ}\cdot m^{-2}\right)$	$G_{ft}\left(\mathrm{kJ}\cdot m^{-2}\right)$	$G_{fc}\left(\mathrm{kJ}\cdot m^{-2}\right)$			
2.0	218	104			

**Table 4:** Comparison of the test and numerical simulation.

	Ultimate Bearing Strength (MPa)			2% Offset Bearing Strength (MPa)		
	Test	Simulation	Error *	Test	Simulation	Error *
DJ-A	560.50	578.94	−3.29%	542.20	570.58	−5.23%
DJ-B	562.36	546.83	2.76%	534.33	542.29	−1.49%
DJ-C	564.40	557.93	1.15%	561.40	557.67	0.66%

* Error = (Test − Simulation)/Test × 100%.

**Table 5:** Peak stress of each ply angle and the number of each layer in the DJ-A model under 6 kN external load. The clearance is 0 μm, and the bolt-tightening torque is 1.8 N·m.

	Ply Angle	Radial Peak Stress (MPa)	Ply Number	Tangential Peak Stress (MPa)	Ply Number	Through-Thickness Peak Stress (MPa)	Ply Number
Upper laminate	−45°	−1253.22	No.21	1018.05	No.21	−104.62	No.17
	90°	−547.24	No.24	673.80	No.24	−67.76	No.2
	45°	−1370.35	No.23	1028.53	No.23	−93.54	No.3
	0°	−1367.04	No.22	1236.67	No.22	−123.04	No.16
Lower laminate	−45°	−1007.24	No.5	635.70	No.5	−52.66	No.25
	90°	−468.67	No.2	517.79	No.2	−47.93	No.13
	45°	−997.04	No.3	701.79	No.7	−52.80	No.11
	0°	−1053.13	No.4	835.62	No.3	−51.74	No.4

**Table 6:** Peak stress of each ply angle and ply number when the external load is 6 kN, the clearance remains 0 μm, and the bolt-tightening torque is 5.4 N·m.

	Ply Angle	Radial Peak Stress (MPa)	Ply Number	Tangential Peak Stress (Mpa)	Ply Number	Through-Thickness Peak Stress (Mpa)	Ply Number
Upper laminate	−45°	−1216.93	No.21	1013.53	No.21	−114.00	No.17
	90°	−532.98	No.24	700.77	No.24	−85.18	No.2
	45°	−1369.65	No.23	1039.45	No.23	−119.45	No.3
	0°	−1385.49	No.22	1266.79	No.22	−119.62	No.16
Lower laminate	−45°	−920.24	No.5	576.90	No.5	−52.22	No.25
	90°	−453.72	No.2	419.61	No.2	−45.34	No.13
	45°	−917.18	No.3	652.02	No.7	−47.30	No.7
	0°	−958.37	No.4	742.63	No.4	−49.03	No.4

**Table 7:** Comparison of the peak stress at a bolt-tightening torque of 5.4 N·m without clearance and at a bolt-tightening torque of 1.8 N·m with an external load of 6 kN.

	Ply Angle	Radial Peak Stress Difference	Tangential Peak Stress Difference	Through-Thickness Peak Stress Difference
Upper laminate	−45°	−2.90%	−0.44%	8.97%
	90°	−2.61%	4.00%	25.71%
	45°	−0.05%	1.06%	27.70%
	0°	1.35%	2.44%	−2.78%
Lower laminate	−45°	−8.64%	−9.25%	−0.84%
	90°	−3.19%	−18.92%	−5.40%
	45°	−8.01%	−7.09%	−10.42%
	0°	−9.00%	−11.13%	−5.24%

**Table 8:** Peak stress of each ply angle and ply number when the external load is 6 kN, the bolt-tightening torque remains 1.8 N·m, and the clearance is 180 μm.

	Ply Angle	Radial Peak Stress (MPa)	Ply Number	Tangential Peak Stress (MPa)	Ply Number	Through-Thickness Peak Stress (MPa)	Ply Number
Upper laminate	−45°	−1164.09	No.25	777.15	No.25	−86.44	No.17
	90°	−1044.92	No.24	913.51	No.24	−67.61	No.2
	45°	−2341.32	No.23	1756.09	No.23	−99.53	No.3
	0°	−2079.97	No.22	1377.03	No.22	−103.21	No.18
Lower laminate	−45°	−1057.99	No.1	613.82	No.5	−40.86	No.1
	90°	−311.20	No.2	537.24	No.2	−20.45	No.13
	45°	−1053.36	No.3	847.04	No.3	−29.16	No.7
	0°	−1449.25	No.4	1025.82	No.4	−26.81	No.10

**Table 9:** Comparison of peak stresses between 180 μm clearance and 0 μm clearance under 6 kN external load and constant bolt-tightening torque.

	Ply Angle	Radial Peak Stress Difference	Tangential Peak Stress Difference	Through-Thickness Peak Stress Difference
Upper laminate	−45°	−7.11%	−23.66%	−17.38%
	90°	90.94%	35.58%	−0.22%
	45°	70.86%	70.74%	6.40%
	0°	52.15%	11.35%	−16.12%
Lower laminate	−45°	5.04%	−3.44%	−22.41%
	90°	−33.60%	3.81%	−57.33%
	45°	5.65%	20.70%	−44.77%
	0°	37.61%	22.76%	−48.18%

## Data Availability

The data presented in this study are available on request from the corresponding author.
